# Rates and risk factors associated with the progression of HIV to AIDS among HIV patients from Zhejiang, China between 2008 and 2012

**DOI:** 10.1186/s12981-015-0074-7

**Published:** 2015-09-25

**Authors:** Lin Chen, Jiezhe Yang, Renjie Zhang, Yun Xu, Jinlei Zheng, Jianmin Jiang, Jun Jiang, Lin He, Ning Wang, Philip Chun Yeung, Xiaohong Pan

**Affiliations:** Zhejiang Provincial Center for Diseases Control and Prevention, Hangzhou, China; National Center for AIDS/STD Control and Prevention, China CDC, Beijing, China; Department of Surgery, Faculty of Medicine, The Chinese University of Hong Kong, Shatin, Hong Kong; Department of HIV/AIDS Prevention and Control, Zhejiang Provincial Center for Diseases Control and Prevention, 3399 Binsheng Road, Hangzhou, Zhejiang People’s Republic of China

**Keywords:** HIV, AIDS, HAART

## Abstract

**Objectives:**

The objective of this study was to determine the rate of acquired immune deficiency syndrome (AIDS) in Zhejiang province and to identify specific factors associated with progression of this disease.

**Methods:**

This study utilized a retrospective cohort to identify the specific factors involved in the progression of human immunodeficiency virus (HIV) to AIDS. We collected data of patients existing in care between 2008 and 2012 from the national surveillance system databases. We performed our analyses using a multivariate Cox proportional hazards model.

**Results:**

This study included 9216 HIV-positive patients (75.6 % male), which yielded 12,452 person-years (py) of follow-up-data. The AIDS progression rates were 33.9 % (2008), 33.6 % (2009), 38.1 % (2010), 30.6 % (2011) and 25.9 % (2012). We observed a significant reduction in the rate of progression Of HIV to AIDS post-2010 (Pearson χ^2^ = 4341.9, *P* < 0.001). The cumulative AIDS progression incidence rates were 33.4, 35.4, 36.4, 37.0 and 37.04 per 100 py in 1 each of the 5 years of follow-up. This study found that age was an independent risk factor for the progression of HIV to AIDS. Compared with patients infected with HIV by homosexual transmission, patients infected with HIV by heterosexuals transmission or blood transfusion had a reduced hazard ratio (*HR*) for progression to AIDS (heterosexual transmission: *HR* = 0.695, 0.524, *P* = 0.007; blood transfusion: HR = 0.524, *P* = 0.015). Diagnosed with HIV from 2011 to 2012 and having a higher CD4+ cell count (350–500 cells/mm^3^; or >500 cells/mm^3^) at baseline were independently associated with lower rates of HIV progression to AIDS [HR = 0.382, 0.380, 0.187, *P* < 0.001]. Patients with a CD+ T-cell count of 200–350 cells/mm^3^ or greater than 350 cells/mm^3^ were less likely to develop AIDS following HIV diagnosis than were those patients without HAART treatment.

**Conclusion:**

This study found a high progression rate from HIV to AIDS in HIV patients residing within Zhejiang province from 2008 to 2010. This rate decreased after 2010, which coincided with the new criteria for HAART treatment, which likely contributed to the observed reduction in the rate of progression. Initiation of HAART with higher CD4+ T-cell count may reduce rate of AIDS progression. Based on our results, we conclude that efficient strategies for HIV screening, as well as early diagnosis and treatment are necessary to reduce the progression of HIV to AIDS.

## Background

Human immunodeficiency virus (HIV), the causative agent of acquired immune deficiency syndrome (AIDS), has posed a major global threat since its discovery in 1983 [1]. Today, approximately 40 million people globally are infected with HIV. Specifically, in China, there is a steadily increasing rate in the prevalence of HIV and subsequent progression to AIDS [2, 3]. In 2009, AIDS became the leading cause of death due to infectious agents in China [4]. Prior to 2009, the People’s Republic of China (PRC) launched the Four Frees and One Care’ (FFOC) policy in 2003 to reduce the transmission of HIV. ‘Four Frees’ represent free antiretroviral treatment, prevention of mother-to-child transmission, voluntary counselling and testing, schooling for Children orphaned by AIDS’ [5]. ‘One care’ represents care and economic assistance to the households of people living with HIV/AIDS. In 2010, the criteria for highly active antiretroviral therapy (HAART) was broadened from standards that included patients with a CD4+ T-cell count ≤200 cells/mm^3^ to those that included patients with a CD4+ T-cell count ≤350 cells/mm^3^. This modification allowed a greater number of people living with HIV (PLHIV) receive free treatment at an earlier stage of the infection. Zhejiang province, located on the southern coast of China, is one of the most developed areas in China. In 2012, there were a total of 8144 HIV/AIDS patients in Zhejiang. Interestingly, Zhejiang has a higher rate of HIV/AIDS patients than other Chinese provinces with similar populations and economic levels [6, 7]. Although 57.0 % of the total patients in Zhejiang have been treated with HAART.

The number of newly diagnosed HIV cases has steadily increased between 2008 and 2012 (from 1066 to 2744 patients). Despite HAART treatment, the number of HIV/AIDs patients in Zhejiang has increased. Therefore, it is essential that we understand the efficacy of current HIV/AIDS management strategies so that we can develop supplementary measures that will delay or prevent the progression of AIDS and, ultimately, reduce the rate of mortality.

Previous studies involving mathematical prediction models suggest that HIV-infected patients to in developing countries are more likely to acquire AIDS and subsequently die compared with those in developed countries [8–10]. In this study, we retrospectively analyzed data from HIV-infected patients in Zhejiang collected from 2008 to 2012. The goal of this study was to utilize patient data to identify risk factors associated with AIDS progression. Because China is the largest developing country in the world, this study examined whether the risk factors identified in developed countries might also be responsible for AIDS progression in developing countries.

## Methods

### Data source

A retrospective cohort study was conducted to evaluate the progression of HIV to AIDS in HIV-infected patients from Zhejiang, China. We utilized patient data from the Chinese National Case Reporting System collected from January 1, 2008 to December 31, 2012. After patients had been positively confirmed as HIV-positive, a uniform questionnaire designed by the Chinese Centers for Disease Control was used to collect patient information. The data were then uploaded to the National Surveillance System Database and the National Treatment Database. Patient information is confidential and could not be released without permission. Information retrieved from these databases included the following: demographic characteristics, disease progression (HIV or AIDS), year of confirmed diagnosis, method of diagnosis, transmission mode, dates, as well as details regarding AIDS progression and treatment [e.g., date of AIDS onset, HAART treatment dates, death (if applicable) CD4+ T-cell count]. After diagnosis, patients were followed until they died, were lost from the database, or until December 31, 2012. Observation stops once AIDS were developed.

### Sampling method

Between January 1, 2008 and December 31, 2012, a total of 9216 HIV-positive patients were enrolled in Group One. All participants had to meet the following criteria: diagnosed as HIV-positive before 2008 and alive on December 31, 2007 or newly diagnosed with HIV during 2008 to 2012; HIV-positive status was defined as testing positive for an HIV-1 on ELISA and Western blot analysis. During the 5 years study observation, 441 (4.8 %) subjects died or dropped out of the database.

Our analysis also focused on the role of potential risk factors in HIV progression to AIDS. Therefore, Group Two consisted of patients with the following characteristics: newly diagnosed as HIV-positive from 2008 to 2012, had CD4+ T-cell count tested within 3 months after diagnosis, no immediate progression to AIDS. Of the 9216 patients, 4121 had AIDS at the time of HIV-positive diagnosis to AIDS. Patients who developed AIDS within 6 months after HIV diagnosis, or patients without a CD4 test within 3 month after diagnosis were excluded in the study to reduce bias. Thus, 3898 patients were enrolled in group two.

### Diagnosis of AIDS

In 2006, the World Health Organization (WHO) stated the progression of HIV to AIDS has occurred when an individual has a CD4+ T-cell count less than or equal to 200 cells/mm^3^, or WHO stage 3 or 4 [11].

### Statistical analysis

The survival time of those patients diagnosed with AIDS was estimated by the number of years from their first visit to the midpoint between the last visit with AIDS-free and the first visit with AIDS. Individuals who remained AIDS-free were monitored until their last follow-up visit. All statistical analyses for this report were conducted using SPSS 17.0 statistical analysis software package (SPSS, Chicago, IL, USA). Data from Group One were analyzed using an abridged life table method, which described the incidence of AIDS from 2008 to 2012; and estimate the effect of HAART treatment on patient outcomes. For Group Two, Cox proportional hazards models were fitted to examine the effects of region, gender, age, education, marriage, occupation, CD4+ T-cell count and HAART treatment on the time to between HIV diagnosis and AIDS diagnosis. Results with p values of less than 0.05 were considered statistically significant. We excluded patients with missing data from the analysis.

## Results

### Patient characteristics

The mean age of the 9216 HIV-positive patients enrolled in Group One was 35 years (range = 1–88 years). In this study, the majority of patients were men (75.6 %) and 45.3 % were married. In terms of educational level, approximately 69.0 % patients did not proceed beyond junior high school. Additionally, 47.4 % patients were migrants from other provinces and most patients held one of the following jobs:worker, famer and commercial servant (64.3 %). Patients demographic characteristics are presented in Table 1.

**Table 1:** Selected characteristics of patients with HIV infection in Zhejiang province

Characteristic	Number (total = 9216)	%
Sex		
Male	6961	75.6
Female	2242	24.4
Occupation		
Farmer	1788	19.4
Worker	2365	25.7
Unemployed	994	10.8
Commercial service	1764	19.2
Doctor, teacher, retired people, government officials	526	5.7
Others	1766	19.2
Marriage		
Unmarried	3396	36.9
Married	4166	45.3
Divorced or widowed	1588	17.3
Unknown	53	0.6
Ethnic group		
Han	8548	92.9
Other	651	7.1
Unknown	4	0.0
Education		
Primary school or under	2461	26.7
Junior high school	3879	42.1
Senior high school	1634	17.8
College or above	1226	13.3
Unknown	3	0.0
Age (years)		
0–24	1964	21.3
25–34	3196	34.7
35–44	2215	24.1
45–54	993	10.8
55 or above	835	9.1
Census register		
Zhejiang Province	4837	52.6
Other Province	4366	47.4

### AIDS development from 2008 to 2012

A total of 9216 patients in Group One were followed up for 12,452 person-years (py). By December 31, 2012, 3868 patients progressed from HIV to AIDS in Group one. There were 441 patients who returned to their home country or died (ranging from 0.30 to 4.70 person-years following HIV diagnosis). Following HIV diagnosis, the mean CD4+ T-cell count was 437 cells/mm^3^. No significant differences between the demographic characteristics of patients who stopped being monitored during the time of our study compared with those patients whose information was available throughout the study time frame (data not shown) (Table 2).

**Table 2:** AIDS progression rates by time period

Time period	Person-years of observation (py)	Incidence observed (n)	AIDS progression rate (/100 py)	Male		Female	
				AIDS, n (py)	AIDS progression rate (/100 py)	AIDS, n (py)	AIDS progression rate (/100 py)
2008	1093	370	33.9	243 (714)	34.0	127 (379)	33.6
2009	1626	546	33.6	395 (1112)	35.5	151 (514)	29.4
2010	2296	874	38.1	676 (1618)	41.8	198 (678)	29.2
2008–2010	5015	1790	35.7*	1314 (3444)	38.2*	476 (1571)	30.3*
2011	3163	969	30.6	726 (2317)	31.3	243 (846)	28.7
2012	4274	1109	25.9	869 (3232)	26.9	240 (1042)	23.0
2011–2012	7437	2078	27.9*	1595 (5549)*	28.8*	483 (1888)	25.6*

* Pearson χ^2^ test, P value for AIDS progression rates 2008–2010 vs. 2011–2012 of whole group, male and female are 0.000, 0.000, 0.002, respectively

During the entire observation period (2008–2012), HIV-positive patients progressed to AIDS (Group one) at the following rates: 33.9 % (2008), 33.6 % (2009), 38.1 % (2010), 30.6 % (2011) and 25.9 % (2012) (Table 2). The cumulative AIDS incidence probability were 33.43, 35.37, 36.38, 36.96 and 37.04 % in 1–5 years of follow-up, respectively (Table 3).

**Table 3:** Cumulative AIDS incidence between 2008 and 2012

Time to AIDS onset (year)	AIDS (n)	Incidence probability (%)	Cumulative incidence probability (%)
0–	2763	33.43	33.43
1–	335	5.81	35.37
2–	151	2.84	36.38
3–	81	1.59	36.96
4–	12	0.24	37.04
Total	3342	–	–

### AIDS progression in patients treated with HAART

**Fig. 1:**
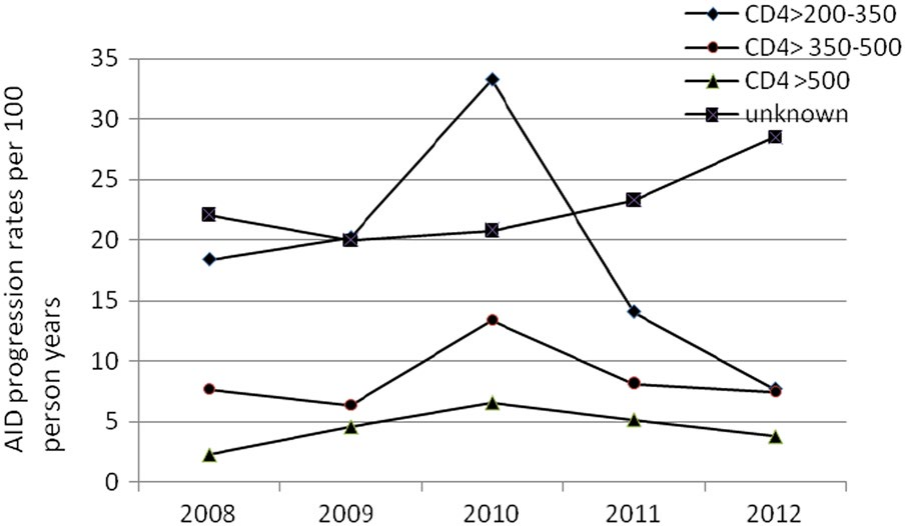
AIDS progression rates by baseline CD4+ cell count among 2008–2012: this *figure* demonstrated the progression of HIV to AIDS among patients with different CD4+ T-cell count

**Fig. 2:**
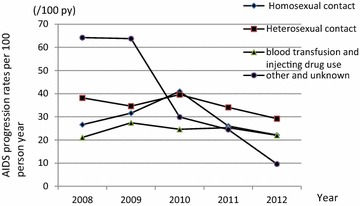
AIDS progression rates by mode of transmission among 2008–2012: this *figure* demonstrated the progression of HIV to AIDS among patients with different transmission routes

The prevalence of AIDS in Zhejiang from 2008 to 2010 was 35.7 %, and it decreased to 27.9 % between 2011 and 2012 (Table 2). To initially qualify for treatment with HAART (2008–2010), patients needed a CD4+ T-cell count of less than or equal to 200 cells/mm^3^; however, the eligibility for HAART treatment was broadened to include patients with a CD4+ T-cell count of less than 350 cells/mm^3^ (2011–2012) (Table 2). Figure 1 show that AIDS progression rates decreased in all CD4 level groups except the unknown one. The largest reduction in the rate of AIDS progression was observed in patients with a baseline CD4+ T-cell count of 200–350 cells/mm^3^ (25.8/100 py of the patients in 2008–2010 and 17.7/100 py of the patients in 2011–2012, P < 0.001). Figure 2 indicates that the progression rates for all modes of transmission decreased after the eligibility criteria for HAART were broadened to include patients with a CD4+ T-cell count of less than 350 cells/mm^3^. In particular, patients who were infected by HIV through homosexual transmission showed the most dramatic reduction in the rates of AIDS (36.2/100 py in 2008–2010 to 23.6/100 py in 2011–2012, *P* < 0.001).

### Changes over calendar time

The demographic characteristics of patients changed over the course of this study, from 2008 to 2012. In 2012, patients with first positive HIV test had a higher median age (39.8 years) compared with that of the comparable group of patients in 2008 (37.9 years old) (*P* < 0.05); HIV infection from homosexual transmission occurred more frequently in patients in 2012 (36.4 %) compared with in 2008 (18.8 %) (*P* < 0.001); However, baseline CD4 T-cell count counts were similar in 2008 and 2012 (median: 319 and 328 cell/μl, respectively; *P* > 0.05); The rate of HAART treatment during the first year of diagnosed with HIV was significantly higher (27.5 %) in 2012 than in 2008 (3.1 %) (*P* < 0.001).

### Risk factors involved in the progression of HIV to AIDS

Patients in Group Two (3989 patients) were followed up for 12,452 py. As of December 31, 2012, it was known that 611 patients had progressed to AIDS. Cox proportional hazards regression was performed to determine the relative contribution of each factor (i.e., demographic characteristics, mobility, transmission routes, year of diagnosis, treatment with HAART and CD4+ T-cell count at baseline) to the progression of HIV of AIDS (Table 4). Cox regression analysis revealed that several factors, including age at diagnosis, transmission routes, census register, year of diagnosis, baseline CD4+ T-cell count and CD4+ T-cell count of initial HAART played significant influence roles in the progression of HIV to AIDS. HIV patients older than 55 years showed a higher risk of progressing to AIDS than patients who were younger than 25 years old (*HR* = 1.915, 95 % CI: 1.357–2.703, *P* < 0.001). The rate of AIDS progression was significantly higher among those infected by homosexual transmission than among patients infected by heterosexuals transmission (*HR* = 0.695, 95 % CI: 0.571–0.844, *P* < 0.05) or by blood borne transmission (*HR* = 0.524, 95 % CI: 0.336–0.816, *P* < 0.05). Patients diagnosed with HIV between 2011 and 2012 were at a lower risk of acquiring AIDS than were those patients diagnosed between 2008 and 2010 (*HR* = 0.382, 95 % CI: 0.302–0.483, *P* < 0.001). Our results also indicate that a higher CD4+ T-cell count was associated with a lower rate of AIDS progression (*HR* = 0.380, 0.187, *P* < 0.001). Patients with a CD4+ T-cell count of 200–350 cells/mm^3^ or greater than 350 cells/mm^3^ were less likely to develop AIDS following HIV diagnosis than were those patients without HAART treatment (*HR* = 0.225, 0.168, *P* < 0.001) (Table 4).

**Table 4:** Cox proportional hazards model of risk factors for development of AIDS

Risk factor	Unadjusted relative hazard	*P*	Adjusted relative hazard	*P*
	*HR* (95 % *CI*)		*HR* (95 % *CI*)	
Sex				
Male	1 (reference)		–	–
Female	0.996 (0.827–1.200)	0.966	–	–
Age (year)				
0~	1 (reference)		1 (reference)	
25~	1.176 (0.949–1.457)	0.139	1.221 (0.982–1.518)	0.072
35~	1.310 (1.031–1.666)	0.027	1.364 (1.065–1.746)	0.014
45~	1.556 (1.167–2.075)	0.003	1.692 (1.247–2.297)	0.001
55~	1.950 (1.412–2.694)	0.000	1.915 (1.357–2.703)	0.000
Education				
Primary school and under	1 (reference)		–	–
Junior high school	0.981 (0.798–1.206)	0.856	–	–
Senior high school	0.943 (0.739–1.205)	0.641	–	–
College and above	0.874 (0.673–1.135)	0.312	–	–
Marital status				
Single	1 (reference)		–	–
Married	1.198 (1.007–1.425)	0.042	–	–
Divorced or widowed	1.094 (0.863–1.386)	0.457	–	–
Unknown	1.313 (0.486–3.526)	0.590	–	–
Mode of transmission				
Homosexual contact	1 (reference)		1 (reference)	
Heterosexual contact	1.019 (0.862–1.205)	0.824	0.695 (0.571–0.844)	0.000
Blood	0.789 (0.514–1.212)	0.279	0.524 (0.336–0.816)	0.004
Other and unknown	0.951 (0.470–1.927)	0.890	0.689 (0.336–1.415)	0.311
Testing mode				
VCT consultant	1 (reference)			–
Health checkup	1.027 (0.807–1.306)	0.831	–	–
Seeing doctor for other disease	1.261 (1.006–1.579)	0.044	–	–
Spouse and children of patient, blood donors	1.380 (1.007–1.891)	0.045	–	–
Others	1.112 (0.720–1.718)	0.633	–	–
Census register				
Zhejiang Province	1 (reference)		1 (reference)	
Other Province	0.765 (0.651–0.898)	0.001	0.670 (0.562–0.799)	0.000
Mobility				
Between Provinces	1 (reference)		–	–
Between Cites	0.905 (0.636–1.287)	0.579	–	–
Between County	0.977 (0.777–1.228)	0.840	–	–
Immobility	0.970 (0.795–1.182)	0.761	–	–
Reported time (year)				
2008–2010	1 (reference)		1 (reference)	
2011–2012	0.346 (0.274–0.437)	0.000	0.382 (0.302–0.483)	0.000
Baseline CD4+ T-cell count				
200–350	1 (reference)	0.000	1 (reference)	
350–500	0.464 (0.388–0.556)	0.000	0.380 (0.316–0.458)	0.000
>500	0.262 (0.206–0.332)	0.000	0.187 (0.147–0.239)	0.000
CD4+ T-cell count of initial HAART				
No HAART	1 (reference)		1 (reference)	
≤200	2.166 (1.273–3.685)	0.004	1.165 (0.679–1.999)	0.580
200–350	0.402 (0.320–0.504)	0.000	0.225 (0.178–0.285)	0.000
>350	0.295 (0.110–0.789)	0.015	0.168 (0.063–0.451)	0.000

*VCT* voluntary counselling test

## Discussion

The rate at which HIV progressed to AIDS decreased from 33.9 % in 2008 to 25.9 % in 2012. These rates were greater than those observed in developed countries, such as British Columbia (7 % in 2013), Brazil (6.2 % between 2000 and 2008), but they were similar to the rates in Africa and Asia countries [12–16]. In this study, AIDS was diagnosed within 1 year of HIV diagnosis, which is similar to the diagnostic process in developed countries. The rates at which patients diagnosed with HIV progressed to AIDS within 12 months in developed countries were as follows: Australia: 65.0 %, Canada: 64.0 % and France: 64.8 % [17]. Our study found late diagnosis of HIV in Zhejiang and other Chinese provinces, which probably affects the survival of patients.

Several studies in developed countries have demonstrated that non-HAART treatment, hepatitis B coinfection, tuberculosis coinfection, being older than 50 years of age, having black or brown skin, intravenous drug abuse, lack of schooling as well as a baseline CD4+ T-cell count lower than 500 cells/mm^3^ were predictors of higher rate of AIDS progression [18–20].

The results from our study suggest that increasing the CD4+ T-cell count necessary to begin HAART treatment is an effective way to delay AIDS progression among PLHIV. The overall AIDS progression rate underwent a steady reduction after 2010 (from 38.1 to 25.9 %) as the treatment was available to patients with a CD4+ T-cell count of 350 cells/mm^3^ [21]. Our analysis also indicated that patients with a CD4+ T-cell count greater than 200–350 cells/mm^3^ benefit the most from early HAART treatment. The progression rate for patients with unknown CD4+ T-cell counts increased over time, with 33.5 % of these patients being diagnosed at the time of death. We hypothesize that these phenomena relate to the fact that these specific individuals did not seek care in time. Furthermore, most of these patients were migrants, had lower lever of education, and older. HAART plays an important role in delaying the progression of HIV to AIDS because it can reduce the HIV–RNA concentration and restoring the immune system [22–24]. In recent years, increasing number of researches encouraged early treatment with CD4+ T-cell count less than 500 cells/mm^3^ or more [25–27]. Although the beneficial effects of HAART have been already observed in patients with a CD4+ T-cell count greater than 200 cells/mm^3^, or greater than or equal to 350 cells/mm^3^, developing counties, including Asia and Africa, need to understand other important factors [e.g. economic factors, side effects or serious non AIDS events (ANAEs) like cancer and cardiovascular disease].

Several factors, including mode of transmission, region, age and CD4+ T-cell count at baseline were related to the rate of AIDS progression in HIV-positive patients. A study performed by Hongjing Yan found a higher rate of disease progression from HIV to AIDS among homosexuals compared with among heterosexuals, intravenous drug users (IDUs) or blood donors [8]. Similar results were observed in this study; however, the proportion of late diagnosis in male who have sex with male (MSM) patients was 19.1 %, which was lower than that of heterosexuals (30.9 %) and others (29.7 %) in Zhejiang (data not published). The high rate of AIDS progression among homosexual patients might be a result of other important factors including stigma, psychological characteristics, genetic or molecular characteristics, as well as coinfection with other disease, such as hepatitis; or tuberculosis [28]. Although this information was not collected in our particular patient database, further investigations are warranted. In Zhejiang, the central regions have the highest rate of AIDS development, which might be related to the fact that these regions contain the highest proportions of homosexuals. A study in Hangzhou conducted between 2006 and 2008 indicated that the infection rates of homosexuals from pubs, bathrooms and clubhouses were 3.5, 12.8 and 2.8 %, respectively, all of which were higher than those observed in any other cities in Zhejiang [29, 30]. The results of our study also suggest that age and baseline CD4+ T-cell count were critical factors that determine the rate of progression of HIV to AIDS. Patients who have lower baseline CD4+ T-cell count and were of an older age tended to have a higher %consistent with previously published works [31].

This retrospective cohort study of patients from Zhejiang, provides information on the rate and contributors to the progression of HIV to AIDS. Our observations highlight the impact of late HIV testing and delayed diagnosis of HIV/AIDS in Zhejiang. Previous studies have suggested that an undamaged immune system predicts delayed progression to AIDS [32] and late diagnosis leads to exhaustion of the immune system. Therefore, the use of additional strategies, such as VCT and provider-initiated HIV testing and counseling (PITC), for HIV screening is necessary to delay the progression of HIV to AIDS.

## Limitations

This has a few limitations. As the cohort was not strictly designed, inaccurate follow-up was inevitable. First, the amount of time between HIV diagnosis to AIDS diagnosis might have been exaggerated or underestimated. Second, CD4+ T-cell count, which is an important diagnostic tool for AIDS, was not available for all patients. A large number subjects are excluded in group two for their AIDS status as diagnosis and without CD4+ T-cell count in first 3 month following diagnosis were excluded for confounding variables, which may have introduced selection bias. Although we recommend early HAART treatment, data on side effect or additional information regarding earlier treatment have not been reported. The role of early HAART treatment in patient health will need to be assessed in the future.

## Conclusions

In conclusion, HIV progression to AIDS was affected by the patient’s age at diagnosis, transmission routes and baseline CD4+ T-cell counts. Early HAART treatment in patients with a higher CD4+ T-cell count significantly delayed progression to AIDS in these patients. Thus effective interventions, such as PITC or VCT, should be utilized to target those at high risk. Our results support use of early HAART treatment, an approach that is already recommended by the current WHO treatment guidelines for HIV infection.
